# Using a high-dimensional graph of semantic space to model relationships among words

**DOI:** 10.3389/fpsyg.2014.00385

**Published:** 2014-05-12

**Authors:** Alice F. Jackson, Donald J. Bolger

**Affiliations:** ^1^Laboratory for the Neurodevelopment of Reading and Language, Department of Human Development and Quantitative Methodology, University of MarylandCollege Park, MD, USA; ^2^Program for Neuroscience and Cognitive Science, University of MarylandCollege Park, MD, USA

**Keywords:** graph, computational model of language, similarity, co-occurrence, distribution model

## Abstract

The GOLD model (Graph Of Language Distribution) is a network model constructed based on co-occurrence in a large corpus of natural language that may be used to explore what information may be present in a graph-structured model of language, and what information may be extracted through theoretically-driven algorithms as well as standard graph analysis methods. The present study will employ GOLD to examine two types of relationship between words: semantic similarity and associative relatedness. Semantic similarity refers to the degree of overlap in meaning between words, while associative relatedness refers to the degree to which two words occur in the same schematic context. It is expected that a graph structured model of language constructed based on co-occurrence should easily capture associative relatedness, because this type of relationship is thought to be present directly in lexical co-occurrence. However, it is hypothesized that semantic similarity may be extracted from the intersection of the set of first-order connections, because two words that are semantically similar may occupy similar thematic or syntactic roles across contexts and thus would co-occur lexically with the same set of nodes. Two versions the GOLD model that differed in terms of the co-occurence window, bigGOLD at the paragraph level and smallGOLD at the adjacent word level, were directly compared to the performance of a well-established distributional model, Latent Semantic Analysis (LSA). The superior performance of the GOLD models (big and small) suggest that a single acquisition and storage mechanism, namely co-occurrence, can account for associative and conceptual relationships between words and is more psychologically plausible than models using singular value decomposition (SVD).

## Introduction

How word meaning is represented and how it is acquired has been a fundamental question in cognitive science, as Landauer and Dumais (1997) point out, since the writings of Socrates. One particular notion in lexical semantics that the accumulation of word knowledge occurs incrementally from repeated exposure to words in spoken and written discourse has been articulated from estimates of dramatic vocabulary growth ranging from 1000 to 5000 words during the school years (Nagy and Anderson, 1984; Graves, 1986; White et al., 1990). This is akin to roughly 7 words each day, which is beyond what would be expected from direct instruction of meaning (Nagy and Anderson, 1984). These assumptions of word learning suggest that the representation of word meaning may be more inherently bound to its contextual environment than exist as an abstract form that indexing some set of semantic primitives (see for example, Fodor et al., 1980). That is, word meanings are fluid and dynamic (Bolger et al., 2008; Kintsch and Mangalath, 2011) and depend heavily on context rather than formal definitions (Barsalou, 1987; Rogers and McClelland, 2011). Conceptually speaking, rather than looking up the meanings of words in a mental “dictionary” when words are encountered, the meanings of words are constructed *ad-hoc* in a contextually-constrained manner (Burgess and Lund, 2000). Thus, understanding of a word's meaning and the ability to comprehend and use it fluently corresponds more closely with the words that one associates directly with it, compared to a catalog of abstract semantic features (Bolger et al., 2008; Bolger and Jackson, under review).

The fundamental notion that associations between words drive lexical semantic processing has dominated the field since Meyer and Schvaneveldt (1971) first showed facilitative priming in lexical decision tasks for associated word pairs like *bread-butter* compared to unassociated pairs such as *bread-tree*. Word pairs such as these were pulled from free-association norms (Deese, 1962; Postman and Keppel, 1970; or more recently, Nelson et al., 1998) in which the relationships between words may be semantically related in some conceptual way, for instance, class inclusion (is-A), feature (has-A), and object attribute (is or can) (Collins and Quillian, 1969; Rumelhart and Todd, 1993). For example, Clark (1970) has identified free association responses as generally consisting of antonyms, synonyms, and super- and sub-ordinate relations. However, this may be a function of nouns relative to other parts of speech (Deese, 1962)—other relationships include agent-action, action-object, and modifier-object which may be more greatly influenced by repeated encounters in context. The degree of overlap between such conceptual features has been shown to account for effects of word similarity (Quillian, 1967; Collins and Quillian, 1969; Rosch, 1973, 1975; Smith et al., 1974). Despite these findings, word pairs generated in free association norms do not necessarily overlap in a “taxonomic” fashion in which they share particular features or attributes (e.g., *bread-butter*), rather these “associations” may reflect many types of relationships (including feature overlap) and has been argued to occur as a function of spreading activation in a semantic network (Quillian, 1967; Collins and Quillian, 1972; Collins and Loftus, 1975). The degree to which two words are associated with one another has been shown to predict numerous semantic phenomena from primed lexical decision and naming tasks to similarity judgments and reading comprehension (see Hutchison, 2003 and Neely, 1991 for reviews). Associative relationships between words have been argued to emerge from lexical co-occurrence in the context of discourse (Burgess et al., 1998). In an analysis of the South Florida free association norms (Nelson et al., 2004), Hahn and Sivley (2011) found that the proximity of two words in context (non-adjacent but within 2–4 words) accounted for a substantial amount of variance in the generated associations. However, others have argued against the notion that associations are a direct result of collocation (Mollin, 2009).

At this point, we must clarify that associative relations or associative meaning is often used to refer to the direct product of free association norms. However, the term associative meaning, as we will use it from here on, has also been used to refer to those relationships that are not driven by feature similarity (Chiarello et al., 1990; De Groot, 1990; Shelton and Martin, 1992). From this perspective, we can see the relationship between association and semantic similarity in line with accounts of syntagmatic (collocational) versus paradigmatic meaning. The distinction between semantic similarity (or semantic feature theory) and association has been suggested to reflect two separate systems both mentally and neurally (Glosser and Friedman, 1991; Glosser et al., 1998): (a) lexical network based on co-occurrence, and (b) a semantic network based on feature or categorical similarity (Collins and Loftus, 1975; Fodor, 1983; Mcnamara, 1992). Behavioral evidence for the dual system theory comes from differences in semantic similarity judgments and free association (Nelson et al., 2004; Steyvers et al., 2004) as well as lexical decision and pronunciation tasks (Seidenberg et al., 1984; McKoon and Ratcliff, 1992) in which both lexical associates which have no conceptual relationship (e.g., *needle-haystack*) and conceptually related words (e.g., *bark-pet*) both account for facilitation effects in word processing tasks (Neely, 1991; Plaut, 1995; Perea and Gotor, 1997; Livesay and Burgess, 1998).

The fundamental problem of association strength and semantic (feature) similarity is that the dissociable relationships between words are rarely pure; words that have high associative strength tend to have some categorical or feature overlap and words that are conceptually similar tend to co-occur in context (Hutchison, 2003). Whereas some have argued that the effects of association are driven largely by feature overlap (Chiarello et al., 1990; Lucas, 2000), it is equally arguable that the degree of feature overlap can be accounted for by the co-occurrence of words in context. It is also important to note that there are several alternative dimensions of semantic relatedness. For instance, Osgood's (1957) attempt to capture connotative, relative to denotative meaning, along a number of adjectivial continua (e.g., active-passive, weak-strong, etc.) has accounted for various aspects of comprehension of word senses.

Context-specific or associative relations of words are problematic for certain other types of models, such as cognitive models of semantic knowledge that specify features or categorical organization (e.g., Collins and Loftus, 1975; Mervis and Rosch, 1981), as category models cannot account easily for contextual constraints (Rogers and McClelland, 2011). However, distributional models can more readily account for context-specific aspect of word meaning, as words may co-occur with other words that belong to disparate inter-connected groups that reflect different meanings. A wide variety of computational models have been developed using distributional bases, such as latent semantic analysis (LSA) (Landauer and Dumais, 1997; Landauer et al., 1998), HAL (Lund and Burgess, 1996), COALS (Rohde et al., unpublished manuscript), SOC-PMI (Islam and Inkpen, 2006), and many other variants. These distributional models have met with success at a variety of tasks ranging from synonymy judgment to essay grading (Kakkonen et al., 2005), indicating that the information contained just within distributions of words is sufficient to meet a surprising range of language-related goals. The prominent distribution models such as HAL and LSA are vector space models in which words or contexts are represented as vectors in multidimensional space. Due to the vast number of words and contexts, the immensity of the vector space is necessarily reduced using an algorithm known as singular value decomposition (SVD). While highly effective as a computational tool, it is questionable whether such a process plausibly reflects a psychological process (Kwantes, 2005; Steyvers and Tenenbaum, 2005; Jones and Mewhort, 2007). More psychologically plausible alternatives have been attempted using episodic memory models (Kwantes, 2005), neural network models (Plaut and Booth, 2000; Rohde et al., unpublished manuscript) and more recently with graph models (Steyvers and Tenenbaum, 2005).

In this paper, we introduce another approach, a graph theoretic model, that constructs a semantic network based on the principles laid out in foundational semantic networks (Quillian, 1967; Collins and Quillian, 1969). In these early computer simulations: “each word has stored with it a configuration of pointers to other words in the memory; this configuration represents the words meaning” (Collins and Quillian, 1969, p. 240). In this vein, graphs are methods of representing data and relationships among data using “nodes” and “edges” or “connections.” Connections between nodes have an associated number referred to as “weight.” In the case of a graph model of language, each node may represent a word, and the weight of a connection between two nodes may represent proximity or frequency of co-occurrence. A possible benefit of graph models of language is that the data are not necessarily collapsed or reduced, though reduction is possible. Instead of SVD or similar algorithms needed to reduce high dimensionality models, reduction of complexity in graphs may be executed using clustering nodes, pruning edges, or performing additional analyses that identify some type of relationship and merging the involved nodes.

Graph models that have been used in the literature have varied widely in the target tasks accounted for and the algorithms employed. For instance, one study identified category exemplars using an algorithm that considered each new exemplar candidate's connectivity to previously identified exemplars (Widdows and Dorow, 2002). Another gauged document similarity using a type of sub-graph comparison that compared the entirety of the documents rather than considering individual terms (Tsang and Stevenson, 2010). One promising approach identified “communities” corresponding to word senses using clique analysis, an algorithm commonly applied to social networks (Palla et al., 2005). And yet another attempted to account for development by examining small-world network distributions of semantic networks and semantic growth according to iterative updating of connectivity between words (Steyvers and Tenenbaum, 2005). The MESA model (Collins-Thompson and Callan, 2007) used random walk Markov chains through a graph whose connections represented several different types of word relationships to judge the quality of word definitions, while Hughes and Ramage (2007) used random walk Markov chains on graphs based on WordNet relationships to judge semantic similarity of word pairs. The consistent feature of these graph models is that each study exploits graph-specific properties of the model and graph analysis algorithms to address their chosen tasks.

Graph-structured models provide certain additional relevance to the psychological study of language, largely stemming from the fact that dimensionality of the model is not reduced in any transformative manner. While low-frequency word or low-weight connections may be deleted from a graph model in order to reduce its computational burden, these deletions don't impact any other words or connections. Each node still represents a word and each connection still represents first-order co-occurrence. In contrast, the matrix reduction used in LSA takes a semantic space with many thousands of dimensions and reduces it to a few hundred dimensions, such that vectors within the resulting space do not correspond directly to any specific concepts (hence the “latent” meaning in “latent semantic analysis”). Thus, maintaining dimensionality in a graph model doesn't eliminate information as SVD does. It records the history of language exposure in a straightforward and transparent manner, and allows for easier interpretation of model output because nodes reflect specific words rather than “latent meaning” (Lund and Burgess, 1996; Burgess and Lund, 1997; Audet and Burgess, 1999).

The goal of this paper is to introduce a graph of language distribution model (GOLD) for English that utilizes the frequency or degree of contextual co-occurrence to account for semantic phenomena using psychologically plausible algorithms. From a theoretical perspective, we attempt to determine whether the GOLD model can account for association relative to conceptual or semantic similarity based upon the distribution of co-occurrences between words. Lund et al. (1995) showed that a co-occurrence model (HAL) using high dimensional vector space could capture categorical relations in the vector elements and that these could be used to generally predict priming data from Shelton and Martin (1992) and Chiarello et al. (1990). The authors suggest that the relationship in the first-order co-occurrences is predictive of associative relationships and that second-order co-occurrences are more important for structural semantics. However, given the nature of their vector-space model, and of vector space models in general, the reduction of the co-occurrence structure to vector space does not allow for the statistical regularities to accrue from episodic memory (Kwantes, 2005; Steyvers and Tenenbaum, 2005). By preserving episodic knowledge in the graph, the GOLD model can directly test how patterns of co-occurrence across nodes in the graph determine semantic structure.

It is imperative to note that the construction of the architecture in GOLD is not meant to account for the entirety of semantic understanding. As succinctly stated by Steyvers and Tenenbaum (2005):

“… We argue that there are in fact compelling general principles governing the structure of network representations for natural language semantics and that these structural principles have potentially significant implications for the processes of semantic growth and memory search. We stress from the outset that these principles are not meant to provide a genuine theory of semantics, nor do we believe that networks of word–word relations necessarily reflect all of the most important or deepest aspects of semantic structure. We do expect that semantic networks will play some role in any mature account of word meaning. Our goal here is to study some of the general structural properties of semantic networks that may ultimately form part of the groundwork for any semantic theory.”

### Overview

The GOLD model is composed of a set of nodes for word tokens and edges (or connections) constructed from lexical co-occurrence drawn from a large internet-based forum. Using neural network classifiers, this model's performance was compared to that of LSA, a vector space model, on two tasks of classifying relationship types among words: (a) classifying related and unrelated word pairs, and (b) classifying word pairs that are associated only, similar only, or both similar and associated.

## Materials and methods

### GOLD model

#### Corpus

In an attempt to capture modern language usage, we collected a corpus from comments on the forum website Reddit (www.reddit.com), which is one of the most frequently visited websites on the internet (www.alexa.com). The benefits of using a Reddit comment corpus include naturalistic language use, a wide range of authors, a broad array of topics under discussion, and a vast pool of data. Posts in the most popular subsections of Reddit (enumerated at http://subreddits.org/) were queried roughly daily from October 2012 through February 2013, and threads containing more than 100 comments were collected. Comments were parsed at the “document” level, which consisted of the entire comment thread; the “paragraph” level, which took <p> and <br> tags as paragraph breaks; and the “sentence” level, which used sentence-final punctuation such as periods and exclamation points as delimiters in addition to the paragraph breaks. The GOLD model was constructed based on the paragraph level data, as a compromise between the computational complexity of full-document processing and the limited span of the sentence-level data. A total of 19,646 comment threads were collected, totaling 4,342,302 paragraphs, 97,976,253 words (types), with 431,822 unique words (tokens). Average paragraph length was 22.8 words, with a median of 15 words, minimum length of 1 word, a maximum length of 2013 words, and a standard deviation of 24.5 words.

#### Preprocessing

The corpus was stripped of several classes of letterstrings. Stop words (closed-class words such as *the, and, of;* using NLTK's English 127-word stoplist; Bird et al., 2009) were removed, on the premise that removal of stop words does not impact the output of the network but does dramatically decrease the computational load of network construction and analysis (Bullinaria and Levy, 2012). This removed 50,064,361 tokens (word occurrences), more than half of the corpus. Furthermore, the relationship between corpus collocation and the probability of generating an associate in a free association paradigm is weakest for immediately adjacent words which are statistically likely to be function/closed class items (Hahn and Sivley, 2011). Thus, the removal of function words is not likely to impact the model's predictive ability. Unique strings that did not occur in a large set of words combined from NLTK's word lists (size 755,110) and NLTK's package of WordNet (size 10,771,928) were removed on the premise that these words are not common terms in the language. This step eliminated letterstrings such as *fooooood*, *hasbut*, and *qxt*, and protowords such as *facepalm, derp*, and *awesomesauce*. A surprising 362,202 types (unique words) were removed in this step, for two reasons. First, retaining only words that occur in wordlists is conservative, as many legitimate words were not present in the wordlists (such as *minnesota* and *minecraft*). Second, the internet is rife with creative misspellings, and these strings are more likely to be unique than correct spellings—for example, *someone* may occur with a high frequency but only count as a single unique type, while *sumone, someon, somoen, summone*, etc., will each count as a separate, unique type. Despite the huge number of types removed in this step, these types accounted for only 2,112,017 tokens, or ~2.15% of the corpus. Lastly, strings that occurred only once in the entire corpus (10,592 tokens, such as *osseous* and *monomorphism*) were removed on the premise that very low frequency words will be connected to a very small set of co-occurring words and thus cannot contribute much to the network processing or to psychological meaning.

A final list of 58,901 types remained after preprocessing, composing a corpus of 45,799,875 tokens. The corpus of paragraphs, after preprocessing, had an average of 10.54 words, with a median of 7 words, minimum length of 1 word, maximum length of 1650 words, and a standard deviation of 11.31 words.

#### Constructing the graph

Co-occurrence of words within the preprocessed corpus was calculated by examining each paragraph in turn, pairing every word in the paragraph with every other word, and incrementing the weight of the connection for each word pair by 1. Paragraphs of length = 1 (e.g., “cuuuuuuuuuute” and, mysteriously, “onychomycosis”) were ignored. The total collection of word pairs and connection weights were fed into graph database software (Neo4j, version 1.8.2; Eifrem, 2009) to construct the graph. A total of 58,901 tokens (nodes) and 54,399,032 weighted relationships among those words (edges) were included in the bigGOLD model; the sum of weights in the model totaled around 490 million.

While the appropriate span of a co-occurrence window is a matter of debate (Mollin, 2009), some previous research has found that co-occurrence models constructed from small window sizes tend to outperform those constructed from larger window sizes (Bullinaria and Levy, 2007) Accordingly, the network was reconstructed using a window of size = 1, such that words were only connected to words that occurred immediately adjacent in the cleaned paragraphs. This network included 58,901 nodes and 10,603,851 weighted edges, and is hereafter referred to as “smallGOLD”; the sum of weights in this model totaled around 41 million.

**Figure 1:**
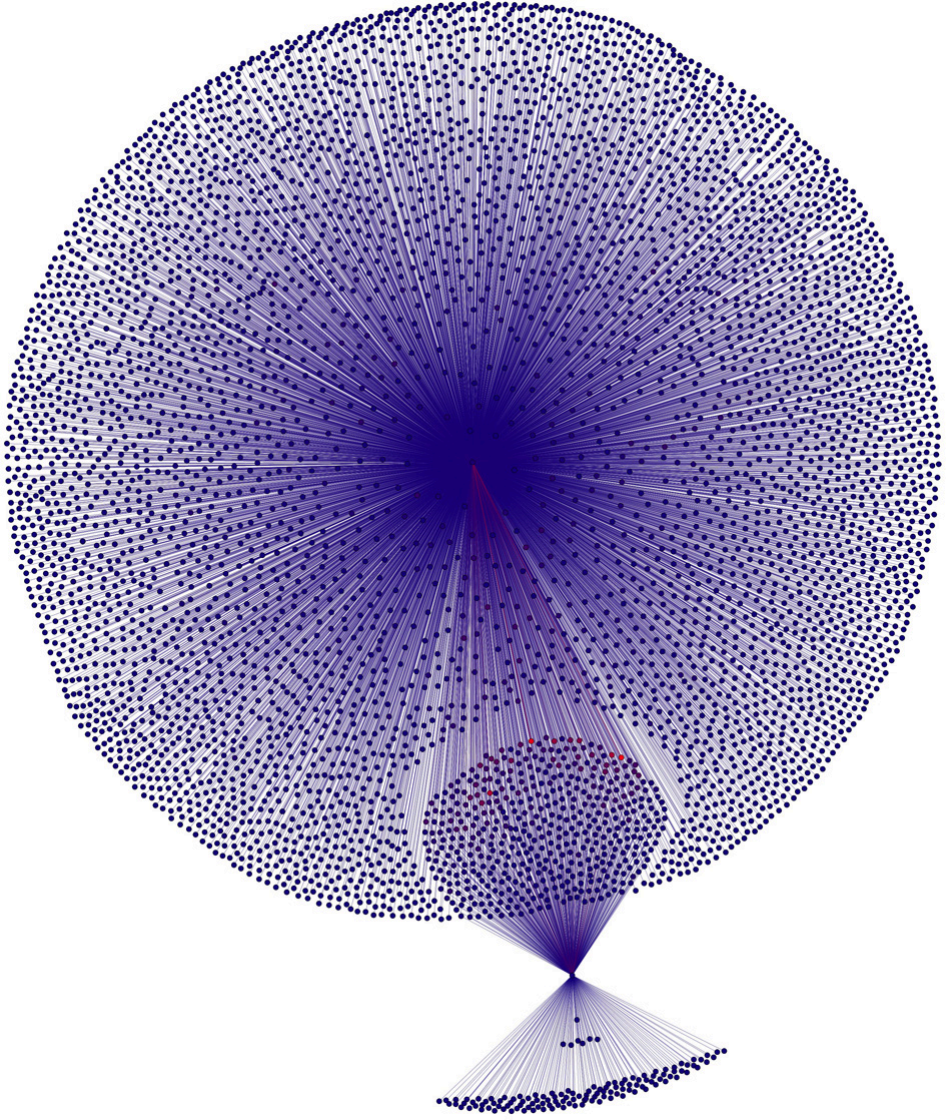
**First-order associates of *grumpy*-*cat.*** Connectivity between associates is not displayed. The large cloud of nodes are the associates of *cat* that are not also connected to *grumpy*; the small cloud of nodes are the associates of *grumpy* that are not also connected to *cat*; and the round blob between them is the set of nodes that is connected to both *grumpy* and *cat*. Figure produced using Force Atlas and Yifan-Hu layout algorithms in Gephi (Bastian et al., 2009).

**Figure 2:**
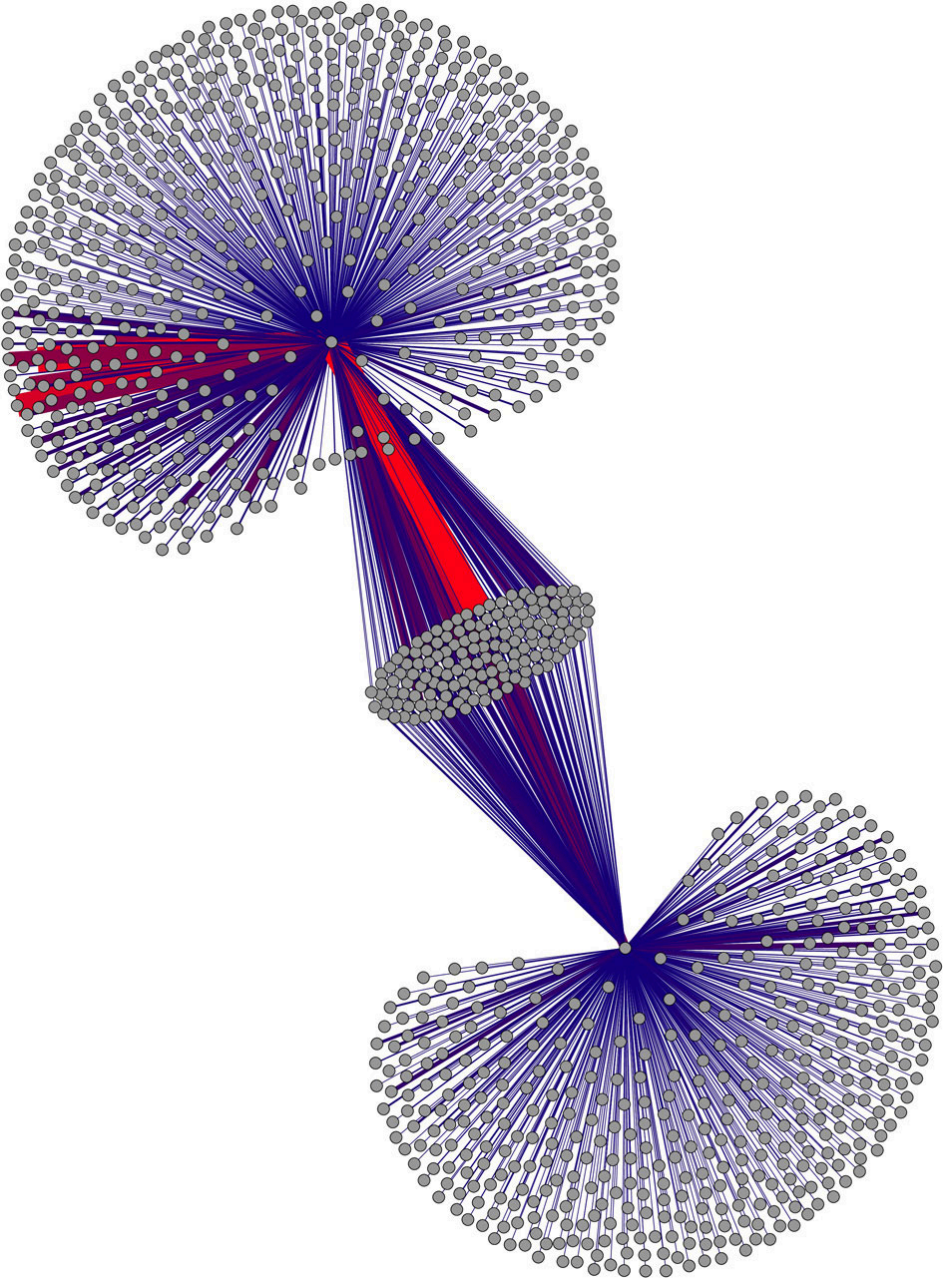
**First-order associates of *sushi* and *octopus.*** Connectivity between associates is not displayed. This subgraph is small enough to display weight information as well; weight of connections is depicted by color (red=large weights) as well as thickness. Figure produced using Force Atlas and Yifan-Hu layout algorithms in Gephi (Bastian et al., 2009).

Figures 1, 2 present first-order connectivity of two pairs of words: *grumpy-cat* in Figure 1, and *sushi-octopus* in Figure 2. The effect of frequency is very apparent in Figure 1, as *grumpy* occurs 754 times in the corpus, while *cat* occurs 17,551 times; accordingly, the size of the *cat* associate cloud dwarfs that of the *grumpy* associate cloud. Figure 2 displays a pair that is much closer in frequency: *sushi* occurs 938 times in the corpus, while *octopus* occurs 512 times. It is worth noting that the higher frequency words are more likely to be in the overlap set (those nodes that are connected to both words of the word pair) merely as a result of frequency.

#### Normalization

High-frequency words carry less information or specificity of meaning than low-frequency words (Finn, 1977; Schatz and Baldwin, 1986). That is, terms with high-specificity are used more rarely (the concept of *antidisestablishmentarianism* doesn't occur often in daily life); inversely, more frequent words tend to be far more polysemous (e.g., run) and as a result is less specific with respect to conceptual reference. In a co-occurrence model, high-frequency words are connected heavily and widely merely as a product of their frequency, rather than reflecting meaningful relationships. Accordingly, these abundant, heavy weights must be normalized to remove this undue influence of frequency. Any method used to normalize these weights must consider the frequencies of the words at both ends of an edge. Several standard methods, such as pointwise mutual information (PMI) and association strength (Van Eck and Waltman, 2009) are already calculated such that they consider the frequencies of both words, while other standard methods that only consider the frequency of a single word, such as inverse document frequency (IDF; Papineni, 2001; Robertson, 2004), may be altered to suit a two-word relationship by combining them in various ways. PMI compares the actual co-occurrence of two words with the co-occurrence that would be expected based on the words' frequencies alone. Document frequency refers to the number of documents (paragraphs) that a word occurs in, while IDF is compares the total number of documents to the number of documents in which a word occurs. The theoretical underpinnings of graph models of language are clear that weights should be normalized, but are not clear on the best manner of normalizing weights. Accordingly, we used 15 different normalization techniques that scale connection weights using various combinations of raw frequency, PMI, document frequency (df), IDF, and log transforms of these frequencies.

#### Similarity and association metrics

Ideal metrics for assessing relatedness between words in the GOLD model should (a) reflect psycholinguistic theories, (b) preferably be limited to a set range of values, such as LSA's -1 to 1, for easy comparison, and (c) differentially consider nodes that are connected to both words in a word pair as well as words that were uniquely connected to each word, as both first- and second-order co-occurrences putatively contribute to relatedness differentially.

Based on assumptions from distribution models (Lund et al., 1995; Landauer and Dumais, 1997), association was theorized to be reflected in the direct connection between the two words in a word pair, which reflects the episodic history of how often the two words co-occur. This metric has no upper bound, and a minimum of 0 indicating no relationship. This metric was calculated by extracting the raw weight of the connection between the two words and normalizing it by the normalization methods in Table 1. An additional metric was determined by calculating PMI as follows, where *w* is the weight between the two words in the word pair, *w*_1_*df* is the document frequency of word 1, and *n*_*docs*_ is the total number of documents in the corpus:

$$PMI=\log_{10}\left(\frac{w*n_{docs}}{w_{1}df*w_{2}df}\right)$$

Additionally, we tested 15 methods of normalizing the connection weights graph-wide (see Table 1 for normalization methods). All permutations of these association algorithms and normalization methods were calculated from the graph, for a total of 30 association metrics (2 association calculation methods × 15 normalization methods).

**Table 1:** **Weight normalization methods**.

**Method #**	**Normalization method**	**Calculation of normalized weight**
1	Raw weights	Weight
2	Pointwise mutual information (PMI)	$\log_{10}\left(\frac{weight*ndocs}{w_{1}df*w_{2}df}\right)$
3	Sum of IDFs	(*w*_1_*idf* + *w*_2_*idf*) ∗ *weight*
4	Product of IDFs	(*w*_1_*idf* ∗ *w*_2_*idf*) ∗ *weight*
5	Sum of document frequencies	(*w*_1_*df* + *w*_2_*df*) ∗ *weight*
6	Product of document frequencies	(*w*_1_*df* ∗ *w*_2_*if*) ∗ *weight*
7	Inverse of sum of IDFs	$\frac{weight}{\left(w_{1}idf+w_{2}idf\right)}$
8	Inverse of product of IDFs	$\frac{weight}{\left(w_{1}idf*w_{2}idf\right)}$
9	Inverse of sum of document frequencies	$\frac{weight}{\left(w_{1}df+w_{2}df\right)}$
10	Inverse of product of document frequencies	$\frac{weight}{\left(w_{1}df*w_{2}df\right)}$
11	Sum of frequencies	(*w*_1_*f* + *w*_2_*f*) ∗ *weight*
12	Sum of frequencies multiplied by log sum of frequencies	$\frac{weight}{\left(w_{1}f+w_{2}f\right)*\log_{10}\left(w_{1}f+w_{2}f\right)}$
13	Product of frequencies multiplied by log product of frequencies	$\frac{weight}{\left(w_{1}f*w_{2}f\right)*\log_{10}\left(w_{1}f*w_{2}f\right)}$
14	Sum of frequencies divided by log sum of frequencies	$\frac{weight}{\left(\frac{\left(w_{1}f+w_{2}f\right)}{\log_{10}\left(w_{1}f+w_{2}f\right)}\right)}$
15	Product of frequencies divided by log product of frequencies	$\frac{weight}{\left(\frac{\left(w_{1}f*w_{2}f\right)}{\log_{10}\left(w_{1}f*w_{2}f\right)}\right)}$

Semantic similarity goes beyond the simple co-occurrence between two words and is theoretically reflected in shared or overlapping patterns of connectivity for two words (Lund et al., 1995), such that two words that are connected to the same community of words with similarly weighted connections are more similar. In essence, the graded nature of similarity (i.e., Collins and Loftus, 1975) might be represented by some combination of the overlapping relative to non-overlapping patterns of connections and the fundamental weighting of those connections. For instance, the words cat and dog will share common nodes for many of the characteristic and defining features of the classes (i.e., animals, mammals, etc.) and categories (i.e., common house pets). These relationships include IS-A (class inclusion) and HAS-A (feature) relations among others that compose the realm of semantic relations. However, the set of features unique to each term (e.g., barks or meows) in relative proportion to the common features putatively determine the gradedness of semantic similarity.

This theoretical conception prescribes the use of unique as well as common features in the assessment of similarity, but does not prescribe the exact calculation of the metric. In order to explore metrics that might be effective in detecting similarity versus association in GOLD, we tested 5 different algorithms described below. All permutations of the similarity algorithms and normalization methods were calculated from the graph, for a total of 75 similarity metrics (5 similarity calculation methods × 15 normalization methods). These metrics are redundant to some degree; however, because one of the primary goals of the present study was to establish if the information necessary to classify stimuli is present in the graph, the full set of metrics was input into the neural network classifiers. This “shotgun approach” is a method of exploring what information may be present in the graph, and allows for exploration into what metrics may be successful. However, it is inappropriate to conclude that any particular metric is “best” based on performance on this limited stimulus set with these limited tasks, because this stimulus set is not designed to span the full space of relationships (e.g., there may be many synonyms and few antonyms in the stimulus set) and thus performance on these tasks may provide an inaccurate view of which metrics are necessary or most predictive.

**Figure 3:**
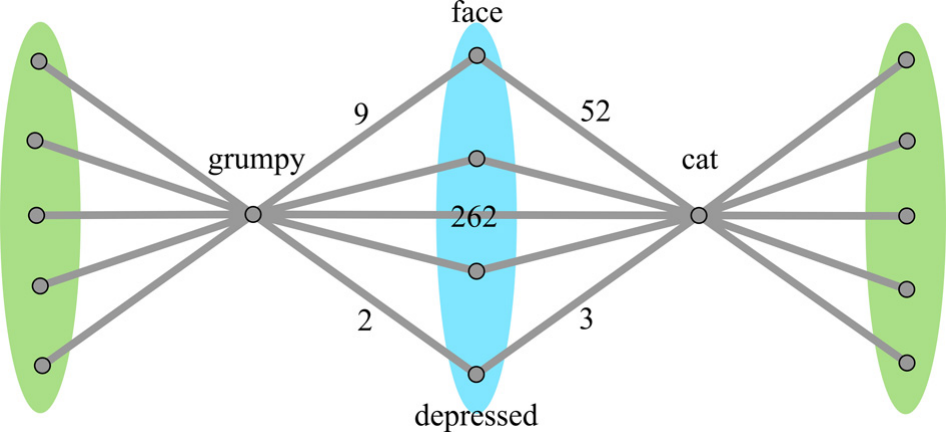
**Simplified graph of associates of *grumpy-cat*.** Nodes on the blue region are the **overlapping** nodes, each of which is connected to both words in the word pair. Nodes on the green regions are the **non-overlapping** nodes, each of which is connected to only one of the words in the word pair. For clarity, only a few nodes are displayed.

***Similarity metric calculation***. Five methods were used to calculate similarity, all considering overlapping nodes and non-overlapping nodes separately. It is theorized that a similar pattern of connectivity to overlapping nodes will arise when the word pair is more similar, but if their connections to non-overlapping nodes are much greater, than the similarity in overlap may not contribute as much to the overall judgment of the word pairs. Accordingly, the following metrics involve various ways of summing weights to the overlapping nodes and summing weights to the non-overlapping nodes, and comparing the two sums. Figure 3 depicts a simplified graph of *grumpy-cat*, with the overlap and non-overlap nodes annotated.

**Method 1:** Overlap and non-overlap sets. The weights to each set are summed as follows, where |Vo| is the number of nodes in the overlap set, |Vn| is the number of nodes in the non-overlap set, and *w*_1_*n*_*i*_ is the weight between word 1 and node *i*:

$$Weights\;to\;overlap=\sum_{i=1}^{\text{|Vo|}}\left(w_{1}n_{i}+w_{2}n_{i}\right)$$

$$Weights\;to\;nonoverlap\;=\sum_{i=1}^{\text{|Vn|}}w_{1}n_{i}+\sum_{i=1}^{\text{|Vn|}}w_{2}n_{i}$$

However, any additive or subtractive combination of these values could be arbitrarily high. It would be ideal if the metric would map to a finite range for easy comparisons (like LSA's output ranges from −1 to 1). One approach is to compare the proportion of the total weights that is accounted for by weights to the overlap and the non-overlap sets. The difference between these proportions will map from −1 (in the case where 100% of weights are connected to non-overlap nodes) to 1 (in the case where 100% of weights are connected to overlap nodes).

Total weights = weights to overlap + weights to nonoverlap

$$Proportion\;to\;overlap=\frac{Weights\;to\;overlap}{Total\;weights}$$

$$Proportion\;to\;nonoverlap\;=\frac{Weights\;to\;nonoverlap}{Total\;weights}$$

Similarity = Proportion to overlap-Proportion to nonoverlap

**Method 2:** Overlap and non-overlap sets, normalized by size. Method 2 is calculated as Method 1, except that *Weights to overlap* and *Weights to nonoverlap* are normalized by their relative sizes, as below:

$$Weights\;to\;overlap\;=\frac{\sum_{i=1}^{\left|\text{Vo}\right|}\left(w_{1}n_{i}+w_{2}n_{i}\right)}{\left|Vo\right|}$$

$$Weights\;to\;nonoverlap\;=\frac{\sum_{i=1}^{\left|\text{Vn}\right|}w_{1}n_{i}+\sum_{i=1}^{\left|\text{Vn}\right|}w_{2}n_{i}}{\left|Vn\right|}$$

The final similarity metric is calculated as in Method 1, as the difference of proportions to the overlap and non-overlap sets.

**Method 3**: Overlap and non-overlap sets, overlap set scaled by magnitude difference. For the remaining methods, the sum of weights to overlap transformed according to the following equation:

$$Weights\;to\;overlap=\sum_{i=0}^{\left|Vo\right|}\left(\frac{w_{1}n_{i}+w_{2}n_{i}}{\left(\frac{\max\left(w_{1}n_{i},w_{2}n_{i}\right)}{\min(w_{1}n_{i},w_{2}n_{i})}\right)}\right)$$

This transform has the effect of scaling the two weights by how close they are in magnitude, such that weights that have a smaller magnitude difference will contribute more of their weight to the final total. In the example in Figure 3, *grumpy-face* has a weight of 9 while *cat-face* has a weight of 52; their combined transformed weight would be 10.56 (18% of the original combined weights). In contrast, *grumpy-depressed* has a weight of 2 while *cat-depressed* has a weight of 3; their combined transformed weight would be 3.33 (66% of the original combined weights).

In Method 3, weights to the overlap nodes are calculated as above, and the final similarity metric is calculated as in Method 1 (no additional normalization).

**Method 4**: Overlap and non-overlap sets, overlap set scaled by magnitude difference, both sets normalized by size. In Method 4, weights to the overlap nodes are calculated as above and then normalized by size as in Method 2. The final similarity metric is calculated as in Method 1.

**Method 5**: Overlap set only, scaled by magnitude difference, normalized by size. In Method 5, only the overlap set is considered, and its weights are calculated as in Method 3 and normalized as in Method 2, as follows:

$$Weights\;to\;overlap=\frac{\sum_{i=0}^{\left|Vo\right|}\left(\frac{w_{1}n_{i}+w_{2}n_{i}}{\left(\frac{\max(w_{1}n_{i},w_{2}n_{i})}{\min(w_{1}n_{i},w_{2}n_{i})}\right)}\right)}{\left|Vo\right|}$$

Because the non-overlap set is ignored, no proportions are calculated. This metric does not map from −1 to 1.

### Latent semantic analysis

LSA is a vector-space model commonly used in language research to gauge word relationships and is often considered the gold standard for performance of a range of measures. Accordingly, LSA was used here as a comparison model. LSA was constructed on the corpus described above using gensim (Rehurek and Sojka, 2004), a Python module. The same preprocessing steps were applied to the corpus and the model was constructed with 300 dimensions, as is often determined to be optimal for LSA model creation (Landauer et al., 1997).

### Word pairs

In order to assess the GOLD model's performance on identifying degree of relatedness between two words and the classification of relatedness as associative or semantically similar, we compared the metrics of the GOLD model against those of LSA on word pairs derived from the extant literature. Word pairs were drawn from Plaut and Booth (2000) and Chiarello et al. (1990). Plaut and Booth's 240 word pairs are categorized as related and unrelated, based on free association norms (Nelson et al., 1998). Chiarello et al.'s 144 word pairs are sorted into three categories according to relationship type: associated only, similar only, and word pairs that are both similar and associated. These categorizations were assigned based on several sets of norms, and the words were balanced on length, frequency, and imageability. These word sets were selected because they differentiate relationship types in different ways, and both have been supported with human subjects data.

### Model predictions

Model predictions were quantified using the Orange machine learning software suite (Demsar et al., 2013). Classifiers were trained with the GOLD metrics and LSA output on (1) the Plaut and Booth word pairs and (2) the Chiarello et al. word pairs. Performance measures were calculated based on 10 iterations of training-testing using a 70/30 random split (i.e., the data were split such that a random 70% of the data were used to train the classifiers, and the remaining 30% of the data were used to test the trained classifiers' performance; this process was repeated 10 times with a different random split each time, and the results of the 10 iterations were averaged).

In keeping with the theme of psychological/neurological plausibility, it seemed appropriate to restrict GOLD's learners to algorithms that are plausibly implementable in a brain. However, what exactly constitutes a psychologically or neurologically plausible mechanism is not clear. Logically speaking, it is the case a neural network of suitable size with one or more hidden layers is capable of performing arbitrarily complex mathematical operations (Hornik et al., 1989); if the brain can operate as the mathematically modeled neural networks do, then it is not obvious that an algorithm like SVM, or even SVD, could not be occurring in the brain. Empirically speaking, realistic models of neurons have found success at modeling a variety of algorithms, including fast Fourier transforms (Velik, 2008) and convolution (Blouw and Eliasmith, 2013). However, for purposes of parsimony, the present study restricted the GOLD predictions to using only neural networks.

In contrast, LSA is not intended to mimic neurological or psychological processes, so we did not limit it to (straightforwardly) neurologically plausible mechanisms. LSA was used as input to several classification algorithms: random forests, CN2, k nearest neighbors, and SVM. Maximal accuracy was achieved with neural networks, so those results are presented here. All classifiers used standard or default parameters within Orange. The neural network used here was a multilayer perceptron (a feedforward network using backpropagation to train) with a single hidden layer (parameters: 1 hidden layer, 20 hidden layer neurons, regularization factor = 1.0, maximum 300 iterations). For further reading on neural networks used in language, see Rumelhart and McClelland (1986).

## Results

### Word pair classification

Performance measures are averaged across 10 iterations of training and testing on randomly selected subsets of the data (70/30 train/test). Overall accuracy per model is presented, as well as tables that display correct performance and errors separated by word category.

We first consider the task of distinguishing related from unrelated words, using the word pairs from Plaut and Booth (2000). On this task, the two GOLD models demonstrated nearly identical, high performance (90% accuracy) (Table 2). Inspection of word pairs that were incorrectly classified (Table 3) reveal that the unrelated words misclassified were sometimes clear errors (*right-found*) but often perhaps related (*split-fight*, *yell-burst, treat-equal*,). GOLD failed to identify some clearly related word pairs (*horse-stall, great-super, take-bring, gives-share, slice-piece, glue-paste, right-wrong, live-death*). It appears that several of these pairs have more specific relationships than relatedness, including synonymy, antonymy, and register differences. LSA performed well (74% accuracy); its most common error was to mis-classify related words as unrelated.

**Table 2:** **Overall classifier accuracy on the Plaut and Booth (2000) related and unrelated word pairs**.

		**Accuracy**
	smallGOLD	90.0%
	bigGOLD	90.4%
	LSA	74.4%

**Table 3:**
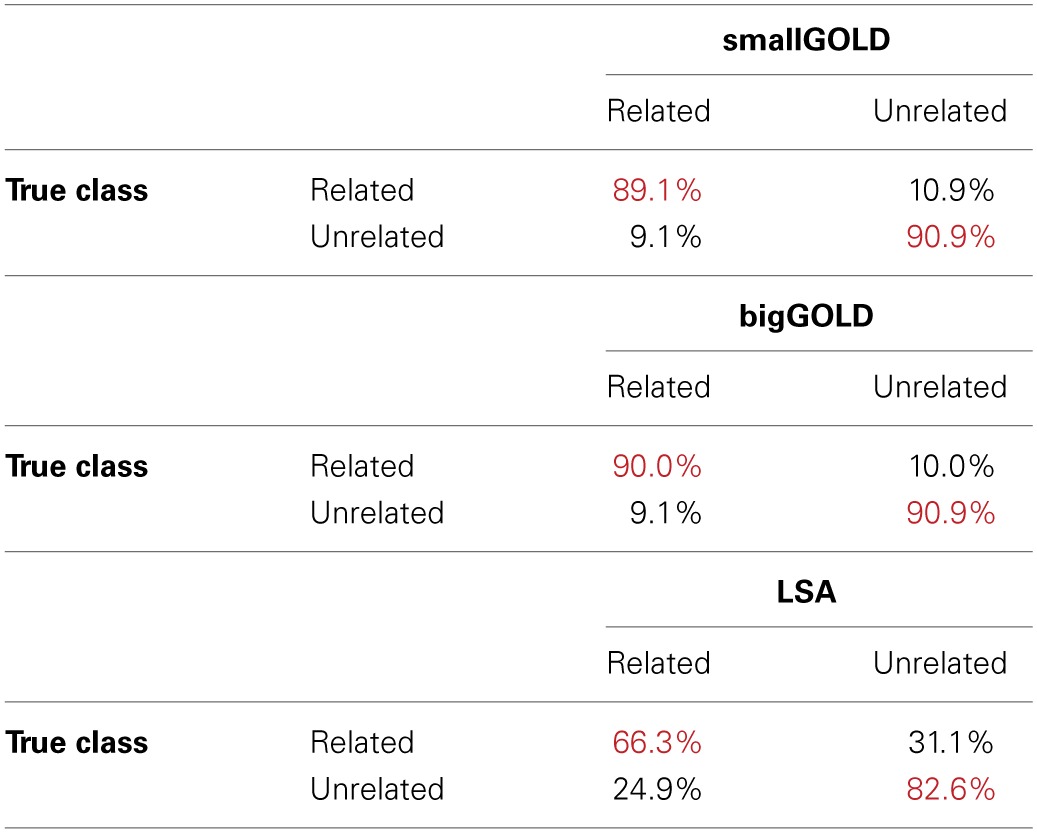
**Classifier performance for the related and unrelated word pairs**.

Percentages shown in red are the correct classifications.

Having established that GOLD can distinguish related from unrelated word pairs, we turn to the task of distinguishing type of relatedness. As stated earlier, the distinction between association and semantic similarity is often a matter of degree as these factors are not orthogonal to one another. Thus, finding word pairs that are stronger in one dimension than the other or are stronger in both is a difficult task. Chiarello and colleagues have identified 144 such word pairs that are semantically related only (or “similar,” e.g., *table-bed*) based upon category membership norms, associatively related only (e.g., *mold-bread*) based upon free-association norms, and both semantically and associatively related (e.g., *aunt-uncle*). Following Lund et al. (1995); Lund et al. (Experiment 3), we tested whether the metrics of the GOLD model could reliably classify these patterns of relationships and compared the results of the GOLD model to those of LSA.

Overall accuracy (Table 4) is best for the smallGOLD model. Inspecting the confusion matrices (Table 5) indicates that the GOLD models' most common error is to mis-classify word pairs that are both similar and associated as associated-only; the next most common mistake is the reverse, where associated-only word pairs are mis-classified as both similar and associated. LSA's most common error is to mis-classify the associated-only words as similar-only. It also assigns similar-only words equally often to the three categories.

**Table 4:** **Classifier accuracy on the Chiarello et al. (1990) associated-only, both associated and similar, and similar-only word pairs**.

		**Accuracy**
	smallGOLD	60.2%
	bigGOLD	57.9%
	LSA	38.8%

**Table 5:**
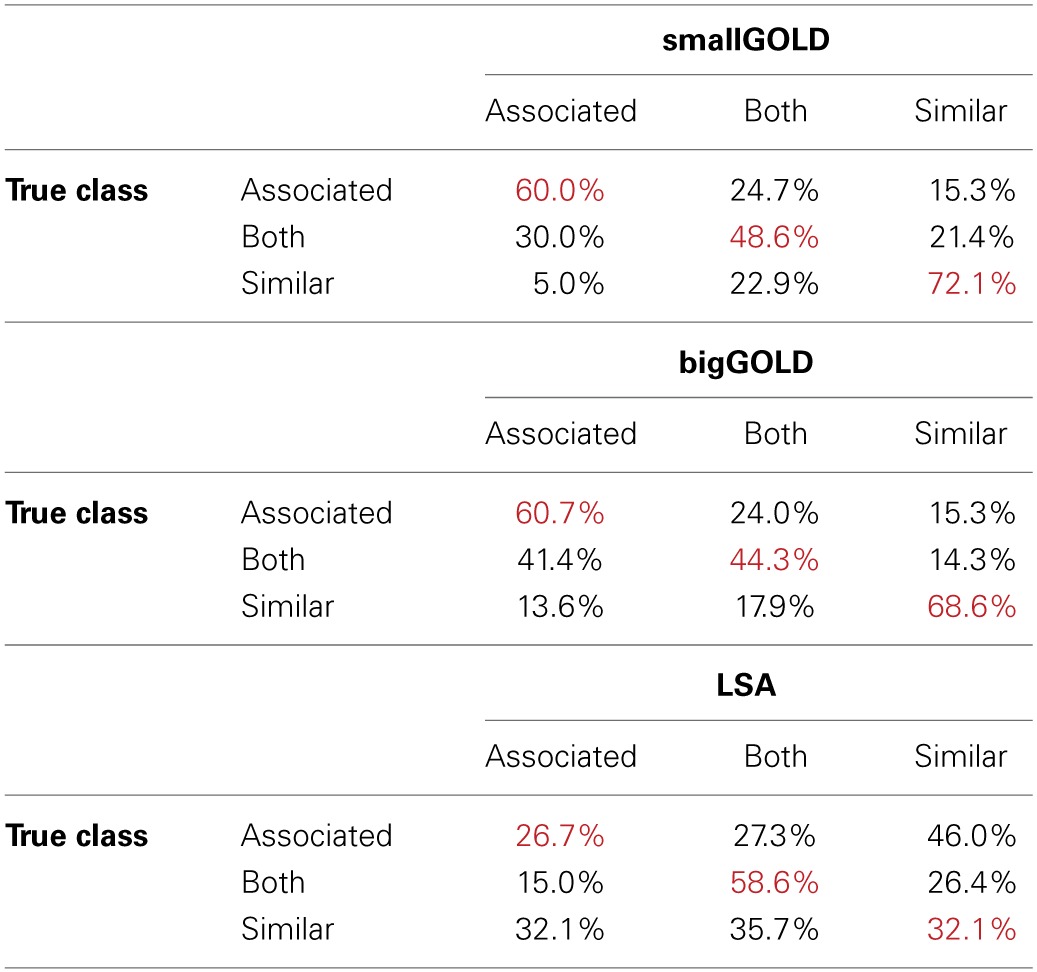
**Classifier performance for the associated-only, both associated and similar, and similar-only word pairs**.

Percentages shown in red are the correct classifications.

### Feature analysis

This exploratory testing of the GOLD model relied on the “shotgun approach” of feature generation, in which all of the combinations of normalization and metric calculation were used as inputs to the neural network. In order to determine which features the algorithm is relying on to produce its classifications, and perhaps to suggest which types of information are important for judging these word relationships, we first compared the performance of similarity metrics to that of association metrics, then investigated individual feature relevance using one- and two-feature classifiers as well as standard feature selection methods.

To compare the utility of each type of metric, two neural network learners classified the similar/associated/both word pair on 5 iterations of 70/30 train/test splits. The first learner was given only the similarity metrics as features, and the second was given only association metrics as features. On the set of related/unrelated word pairs, the classifier using only the similarity metrics reached 47.91% accuracy, while the classifier using only the association metrics reached 55.35% accuracy. On the set of similar/associated/both word pairs, the classifier using only the similarity metrics reached 83.71% accuracy, while the classifier using only the association metrics reached 90% accuracy.

Next, to consider the utility of the different methods of calculation and normalization, features were considered individually and in pairs. For these one- and two-feature classifiers, a neural network learner classified the similar/associated/both word pair on 5 iterations of 70/30 train/test splits. In the first round of analysis, the neural network was given each of the 105 smallGOLD features individually; the maximum accuracy of these 105 classifiers reached 50%. The full set of 105 features was sorted and the 50 highest-accuracy features were retained. In the second round of analysis, the neural network was given all combinations of two features from these 50 features, one pair of features at a time; maximum accuracy reached 63% accuracy, which is on par with the full set of features. Inspection of these feature pairs revealed that the majority of the top ranked pairs included two types of metrics: Method 5 from the similarity metrics (which considered only overlapping nodes, weighted by magnitude difference and normalized by size) and the PMI calculation of association. All but two of the top fifty performers were pairs that included one association and one similarity measure.

Limiting the neural network inputs to those two metrics (Method 5 of the similarity metrics and the PMI calculation of association, totaling 30 pairs of features, shown in Table 6) yielded 63% accuracy. Using additional feature selection (linear SVM weights) to reduce the number of features to 10 produced 65% accuracy; reducing the number of features to 5 boosted accuracy to 68%, which is well in excess of performance using the full set. However, these performance outcomes should be interpreted as exploratory only. The broad conclusion regarding features is that the combination of association (direct connections between the two words) and similarity (based on the overlapping and non-overlapping neighbors of the two words) metrics is more powerful at predicting category than either alone. It may be possible to conclude that the similarity metric considering normalized overlap only and the PMI calculation of association are the most useful, but the similar/associated/both word pairs from Chiarello et al. are not designed to span the language space and thus this finding may not generalize to other regions of the graph or other tasks. Thus, while the use of non-overlapping sets in the case of Method 5 may be more effective for discriminating between these word pairs, yet it may not be as useful in accounting for more graded semantic decisions such as distinguishing between near synonyms or antonyms (e.g., verbal analogies) which may make use of non-overlapping set information.

**Table 6:** **Methods and accuracy of the top 30 pairs of features described above**.

**Rank**	**Type of metric**		**Calculation method**		**Normalization method**		**Acc.**
	**Feat. 1**	**Feat. 2**	**Feat. 1**	**Feat. 2**	**Feat. 1**	**Feat. 2**	
1	Assoc.	Sim.	PMI	Method5	Prod freq ∗ log prod freq	Sum freq/log sum freq	63.23
2	Assoc.	Sim.	PMI	Method5	Weight/(w1df ∗ w2df)	Sum freq/log sum freq	62.58
3	Assoc.	Sim.	PMI	Method5	(w1idf + w2idf) ∗ weight	Sum freq/log sum freq	61.94
4	Assoc.	Sim.	PMI	Method5	Prod freq/log prod freq	Sum freq/log sum freq	61.94
5	Assoc.	Sim.	PMI	Method5	Sum freq ∗ log sum freq	Sum freq/log sum freq	61.94
6	Assoc.	Sim.	PMI	Method5	w1idf ∗ w2idf ∗ weight	Sum freq/log sum freq	61.94
7	Assoc.	Sim.	PMI	Method5	Weight/(w1df + w2df)	Sum freq/log sum freq	59.35
8	Assoc.	Sim.	PMI	Method5	Prod freq ∗ log prod freq	Weight/(w1df + w2df)	59.35
9	Assoc.	Sim.	PMI	Method5	Weight/(w1df ∗ w2df)	Weight/(w1df + w2df)	58.71
10	Assoc.	Sim.	Rel	Method4	Sum freq/log sum freq	(w1idf + w2idf) ∗ weight	58.06
11	Assoc.	Sim.	PMI	Method5	Sum freq/log sum freq	Sum freq/log sum freq	58.06
12	Assoc.	Sim.	PMI	Method5	w1df ∗ w2df ∗ weight	Sum freq/log sum freq	58.06
13	Assoc.	Sim.	rel	Method4	Weight/(w1df + w2df)	(w1idf + w2idf) ∗ weight	57.42
14	Assoc.	Sim.	PMI	Method5	(w1df + w2df) ∗ weight	Sum freq/log sum freq	57.42
15	Assoc.	Sim.	PMI	Method5	(w1f + w2f) ∗ weight	Sum freq/log sum freq	57.42
16	Assoc.	Sim.	PMI	Method5	Sum freq ∗ log sum freq	Weight/(w1df + w2df)	57.42
17	Assoc.	Sim.	Rel	Method4	Weight/(w1df + w2df)	Weight/(w1idf ∗ w2idf)	56.77
18	Assoc.	Sim.	PMI	Method5	(w1df + w2df) ∗ weight	Weight/(w1df + w2df)	56.77
19	Assoc.	Sim.	PMI	Method5	(w1f + w2f) ∗ weight	Weight/(w1df + w2df)	56.77
20	Assoc.	Sim.	Rel	Method4	Sum freq/log sum freq	Raw	56.13
21	Assoc.	Sim.	Rel	Method4	Sum freq ∗ log sum freq	Weight/(w1idf ∗ w2idf)	56.13
22	Assoc.	Sim.	PMI	Method5	(w1idf + w2idf) ∗ weight	Weight/(w1df + w2df)	56.13
23	Assoc.	Sim.	PMI	Method5	Prod freq/log prod freq	Weight/(w1df + w2df)	56.13
24	Assoc.	Sim.	PMI	Rel	Prod freq/log prod freq	(w1idf + w2idf) ∗ weight	56.13
25	Assoc.	Sim.	PMI	Method1	Prod freq ∗ log prod freq	Raw	56.13
26	Assoc.	Sim.	PMI	Method1	Prod freq/log prod freq	Raw	56.13
27	Assoc.	Sim.	Rel	Method4	w1idf ∗ w2idf ∗ weight	(w1idf + w2idf) ∗ weight	55.48
28	Assoc.	Sim.	Rel	Method4	w1idf ∗ w2idf ∗ weight	Raw	55.48
29	Assoc.	Sim.	Rel	Method4	Sum freq/log sum freq	Weight/(w1idf + w2idf)	55.48
30	Sim.	Sim.	Method1	Method5	Prod freq ∗ log prod freq	Sum freq/log sum freq	55.48

## Discussion

### GOLD

The fundamental goal of this paper was not to argue that the GOLD model is a psychological model of word relationships, but rather that as a computational model using more psychologically plausible architecture, the GOLD model could viably account for the relations between words utilizing a graph constructed from the single mechanism of co-occurrences between words in discourse context. As such, the GOLD model performed very well (90% accuracy) on the simpler task of classifying words as related or unrelated. It performed well, but not as well (60%+ accuracy) on the more difficult task of determining whether the Chiarello et al. (1990) word pairs were similar, related, or both similar and related; however, this performance is considered with respect to an LSA model that reached only 39% accuracy on this task. GOLD reached ~60, 50, and 70% on the three relationship categories considered individually, and when it erred, it tended to err on word pairs in the “both” category. This error may reflect model error or may reflect disparate strengths of the two types of relationship—e.g., a given word pair may be strongly similar but only weakly associated, and thus technically be related in both ways, but be misclassified as similar only. Both of the GOLD models were more likely to misclassify “both” items as “associated” than “similar” (bigGOLD. ~41 vs. 14%; smallGOLD, ~30 vs. 21%). The disparity in the misclassifications is greater for bigGOLD, suggesting that the larger window size in bigGOLD may have had an effect on the range of words that were judged to be associated. Both models were also much less likely to classify a word pair with only one relationship type (“associated only” or “similar only”) as the other relationship type; if they erred on these word pairs, they were much more likely to categorize them as “both.”

An alternative explanation for GOLD's misclassifications may not reflect an error in the model, but rather the fundamental difficulty of assigning words to these non-orthogonal categories as Chiarello and colleagues have done. In essence, the GOLD model, using a corpus of more natural language use and preserving that history in the connectivity patterns, may reveal that conceptually related words co-occur more frequently than assumed by research claiming to isolate semantic from associative effects (i.e., Fischler, 1977a,b; Chiarello et al., 1990; Shelton and Martin, 1992; see Lucas, 2000 for review). It may be the case that the question of “how similar are these two words” is ill-posed to some degree. Consider *hot* and *cold:* these words are antonyms, but both are temperatures, and thus perhaps more similar than *hot* and *rutabaga*. *Earthquake* and *tornado* are wildly different concepts, but in a list of *earthquake, tornado*, and *democracy*, suddenly they are much more similar. It may be the case that larger contexts, such as those already used in judgments of document similarity, are necessary for more meaningful judgments of similarity. Future research with the GOLD model should address the development of metrics from GOLD that can be expanded to arbitrary-length inputs, which may enable greater predictive power as well as more accurate modeling of psychological reality.

The smallGOLD and the bigGOLD models performed almost identically on the task of distinguishing related from unrelated words, and while smallGOLD outperformed bigGOLD on the task of classifying types of relatedness, its performance was not drastically better (only ~2% overall). Bullinaria and Levy (2007) suggest that selecting a window size involves a trade-off: larger window sizes may be more susceptible to noise in the form of contexts that don't directly support word meaning, but the larger window size leads to far more co-occurrence data. This may be particularly relevant in bigGOLD, in which the window size was variable and consisted of entire paragraphs. Each of these paragraphs, which ranged in length from one to more than two thousand words—the median paragraph size here is 15 words much shorter than that which are found in expository forms outside of the internet, could include a wide range of words whose meanings may or may not be closely related. However, Bullinaria and Levy (2007) also note that the effectiveness of a given window size is intertwined with other factors, such as task and the metrics used, so it may be the case that the present study's choice of corpus, metrics and specific tasks did not emphasize latent differences between the bigGOLD and smallGOLD models.

From a theoretical perspective, the predictive power of the GOLD model, which was constructed from co-occurrence alone, indicates that the information used to judge relationships among words may be present in lexical co-occurrence. In comparison to models of semantic memory that argue for separable, distinct mechanisms for processing semantic/conceptual similarity and lexical association (Fischler, 1977a,b; Seidenberg et al., 1984; Glosser and Friedman, 1991; Shelton and Martin, 1992), our results suggest that information sufficient to represent both relationship types is present in lexical co-occurrence. This predictive success lends support to a single-mechanism model of word knowledge, and suggests that the method of calculating relationships, rather than representing relationships, may be what differs between relationship types. This is in keeping with theories that word meaning is constructed or retrieved on an *ad-hoc* basis (Kwantes, 2005, see Neely, 1991 for review), as multiple mechanisms of querying may reasonably be involved in that *ad-hoc* construction. Whereas we cannot decisively argue that there are not two unique mechanisms for semantic and associative knowledge, we can suggest that the information necessary to make the types of distinctions between semantically-related and associatively-related words is present in a single graph network constructed from the co-occurrence of words in context. The algorithms for distinguishing between semantic and associative relations are *ad-hoc* computations used to retrieve information from the model. In psychological terms, the necessity for two storage mechanisms is lacking if the same information can be retrieved from a unitary system based on principles of episodic memory formation. Preliminary analysis of the neural network classifier using the GOLD metrics indicates that the combination of association and similarity metrics are more powerful predictors than either type of metric alone, which lends additional support to this multiple querying mechanism account of word meaning.

It is critical to note is that the metrics of GOLD were used in this case to classify words in terms of relatedness and, unlike Lund et al. (1995), these were not simulations of behavioral priming data. As such, it is difficult to say whether the model reflects automatic spreading of activation or post-lexical retrieval processes. It may be the case that both are true of this model if tuned with multiple attractor networks (Plaut, 1995; Plaut and Booth, 2000) in which early state attractors rapidly focus on first-order relations whereas secondary or later stage attractors or statistical computations settle on a topic or gist based representation (Griffiths et al., 2007). Such a model, the *language and situated simulation of conceptual processing* (LASS) model, has been suggested by Barsalou et al. (2008). In this model, the language simulation component, like the surface model in Kintsch's Construction-Integration model (Kintsch and van Dijk, 1978), is driven by automatic lexical associations that enable spreading activation and the establishment of thematic roles from discourse. The situated simulation (like the situation model) is the conceptual level where modality-specific simulations occur from further activation patterns settling into the semantic to enable ultimate comprehension. While the LASS model is inherently a multiple system model as conceptual representations are argued to be grounded in modal (sensory) systems, the nature of activation and retrieval of this latter system may putatively stem from settling of the word level network in a model such as GOLD.

### Graphs

Graphs are a valuable tool in psycholinguistics research, both in service of analysis and of understanding. As a boon to analysis, graphs do not require discarding vast tracts of data in the process of dimensionality reduction, and so the model may maintain a higher degree of complexity that preserves additional information about relationships between words as well as overall statistical regularities that reflect the model's “experience” with language (see Steyvers and Tenenbaum, 2005). However, these benefits, particularly the retained information, are accompanied by a major drawback: computational complexity. Analyzing graphs, particularly very large graphs as one might encounter in a language model, is computationally expensive. The patterns that may prove most interesting are also very complex, such as subgraph isomorphisms, which may be useful for word sense disambiguation or identifying word relationships. Other types of graph theory algorithms may be valuable for identifying language features or word attributes, such as social network analysis for identifies “bridge nodes” that may be homographs, or clique analysis that may be able to cluster register, or connotative/emotional content (Osgood, 1957), or feature similarities (Plaut, 1995; McRae et al., 1997). These algorithms are much more computationally complex than algorithms like SVD, and may require exponentially more time to execute.

One issue in graphs of word co-occurrence is that their high degree of interconnection makes many standard graph algorithms less useful, such as spanning trees and various measures of separation (e.g., Dijkstra's algorithm, Dijkstra, 1959). These algorithms are of course applicable, but may vary in their informativeness because the high degree of interconnectivity in a word-word graph means that words are typically very few steps away from any other word. *n* a graph with this property, the weights of connections are more important than the presence of connections. Accordingly, analyses must focus on algorithms that consider connection weights (Mollin, 2009), algorithms that consider larger patterns of weighted connectivity, or methods of pruning the graph such that the presence of connections becomes informative—perhaps by pruning low weight connections, or limiting words to some arbitrary number of connections. It may also be valuable to maintain more information during the graph construction process. In the present large GOLD model, each connection is weighted with weight = 1, regardless of actual distance between words. It may be useful instead to record connection counts at several distances—e.g., *grumpy* and *cat* co-occur immediately adjacent *n*_0_ times, separated by one word *n*_1_ times, separated by two words *n*_2_ times, etc. Maintaining word order information (perhaps through directional connections) may be a better predictor of human behavior as well, because, for example, *bread-butter* has a higher free association probability than *butter-bread*, etc.

Lastly, as with all models of language, vagaries of the corpus can influence model performance. The corpus from which the GOLD model in the present study was constructed may display a greater influence of conversational speech than, say, textbook-based corpora, as well as unorthodox grammatical structures and word usage. It also has a rather larger vocabulary of obscenities than a corpus constructed from the New York Times might, and spans different topics than standard language corpora (e.g., TASA; see Landauer et al., 1998). It was the aim of this corpus that it span a large range of unadulterated modern language use to provide more ecological validity with respect to the behavioral data to which the GOLD model may be applied.

## Conclusion

The present study constructed a graph model of language (GOLD) from lexical co-occurrence, and used GOLD to predict relationships types among words. The predictive power of the GOLD model, which was constructed from co-occurrence alone, indicates that the information used to judge relationships among words may be present in lexical co-occurrence. GOLD was able to predict multiple varieties of relationships between words (relatedness, similarity, and association), which implies that information sufficient to represent these relationship types is present in lexical co-occurrence. This predictive ability lends support to a single-mechanism model of semantic and associative knowledge, and suggests that perhaps the method of calculating relationships, rather than representing relationships, is what differs between relationship types. Furthermore, the model reached some degree of psychological plausibility in its representation and its use of metrics that are based on theoretical conceptions of word relationships. No higher-order calculations such as SVD are required for extracting relationships from the graph, although complex operations are not necessarily neurologically implausible. In sum, the benefits of using graphs to study language are abundant: the combination of psychological theory- and graph theory-based approaches with data-driven computational methods provides a wealth of novel perspective and analytical approaches.

## Author contributions

This research was conducted as partial fulfillment of the dissertation thesis for Alice F. Jackson. The investigation was designed and conducted by Ms. Jackson with oversight from her research advisor, Donald J. Bolger. Both authors participated in the writing of the manuscript. The authors assert that this research was conducted without conflict of monetary incentive or financial gain.

### Conflict of interest statement

The authors declare that the research was conducted in the absence of any commercial or financial relationships that could be construed as a potential conflict of interest.
